# Effect of non-surgical periodontal treatment on HbA1c levels in non-diabetic patients with chronic periodontitis in an Iranian population

**DOI:** 10.15171/japid.2019.013

**Published:** 2019-12-18

**Authors:** Fereshteh Naseralavi, Masumeh Nikkhah, Nastaran Etemadi, Ashkan Salari

**Affiliations:** ^1^Department of Operative Dentistry, Dental School, Guilan University of Medical Sciences, Rasht, Iran; ^2^Department of Periodontics, Dental School, Guilan University of Medical Sciences, Rasht, Iran; ^3^Dental Student, Dental School, Guilan University of Medical Sciences, Rasht, Iran

**Keywords:** Blood glucose, diabetes mellitus, glycated hemoglobin A, non-surgical periodontal debridement, periodontitis

## Abstract

**Background:**

Evidence is limited on the effect of periodontal treatment on improving HbA1c levels in non-diabetic patients with chronic periodontitis. This study aimed to compare HbA1c levels in non-diabetic patients without periodontitis and nondiabetic patients with chronic periodontitis at baseline and to evaluate the effect of non-surgical periodontal treatment on glycemic control in non-diabetic chronic periodontitis patients.

**Methods:**

In this interventional study, 30 non-diabetics, aged 35‒65 years, were selected and divided into two groups (n=15). Group A consisted of non-diabetics without periodontitis, and group B consisted of non-diabetics with mild to moderate chronic periodontitis. For all the subjects, periodontal parameters, including plaque index, gingival index, periodontal pocket depth, and clinical attachment loss, and laboratory parameters of FBS and HbA1c were measured and recorded. Independentsamples t-test was used to compare periodontal and laboratory parameters between the two groups; paired-samples t-test was used for intra-group comparisons.

**Results:**

HbA1c level in group B (5.4±0.42%) was significantly higher than that in group A (5.04±0.43%) (P=0.03) at baseline. Three months after treatment, improvements were achieved in all the periodontal parameters in group B, with a significant decrease in HbA1c levels (P=0.006).

**Conclusion:**

Non-surgical periodontal treatment resulted in a significant decrease in HbA1c levels in non-diabetic patients with chronic periodontitis. Although these levels did not reach the level of non-diabetic patients without periodontitis, it could be concluded that an improvement in the periodontal condition might lead to near-normal glycemic levels.

## Introduction

Diabetes mellitus comprises a group of metabolic diseases identified by high blood glucose levels (hyperglycemia) and an inability to produce or use insulin.^1^ There are two main types of diabetes: types 1 and 2, while there are other less prevalent and significant types as well. Type 1 diabetes mellitus is characterized by the autoimmune destruction of the insulin-producing β-cells present in the islets of Langerhans, resulting in reduced insulin production. Type 2 diabetes mellitus is caused by environmental resistance to insulin action, impaired insulin secretion, and enhanced glucose production in the liver. Another type of diabetes is secondary hyperglycemia due to some diseases. A typical example of this type of hyperglycemia is gestational diabetes. Other types of secondary diabetes include diabetes associated with the diseases of the pancreas and the destruction of insulin-producing cells. Endocrine diseases, such as acromegaly and Cushing's syndrome, tumors, pancreatitis, and drugs that alter blood insulin levels, are all in this group.^2^

Based on the epidemiological studies conducted in Iran in the last decade, the Iranian diabetic population is estimated at over 1.5 million people.^3^ In addition, there are two types of pre-diabetic conditions, called impaired glucose tolerance and impaired fasting glucose. Individuals with high blood glucose, which is not high enough to be classified as diabetes, are known to have pre-diabetes and are at high risk of developing diabetes in the future.^1^

The effect of diabetes on periodontium has been thoroughly investigated. Perhaps, the most prominent changes in uncontrolled diabetes are a decrease in defense mechanisms and an increased susceptibility to infection, leading to destructive periodontal disease. In fact, periodontitis is one of the complications of diabetes.^4^

Periodontitis is a chronic inflammatory disease characterized by the destruction and loss of connective tissue attachment. Evidence suggests that microbial biofilms and host predisposition play a critical role in the onset and progression of periodontitis.^5^ Recent studies have suggested that chronic periodontitis is a potential risk factor for systemic diseases and low glycemic control in diabetes mellitus.^6-7^

Researchers have linked periodontitis and various diseases or systemic conditions, including diabetes.^8^ In periodontitis, there is the production of proinflammatory mediators, such as interleukin-1β, tumor necrosis factor-alpha, interleukin-6, interferon-gamma, and high levels of acute-phase proteins, such as c-reactive protein increase. All these mediators have a major impact on the metabolism of glucose and fat. TNF-α interferes with fat metabolism and is an insulin antagonist.^9^ IL-6 and IL-1β are also antagonists of insulin activity.^10^ Increased levels of CRP lead to insulin resistance.^11^ IF-γ induces programmed cell death of pancreatic β cells.^12^ Further evidence and observational studies are available on the effect of periodontal therapy on glycemic control in diabetic patients.^13-16^

Evidence suggests that periodontal infection impairs glycemic control in diabetics. Furthermore, effective periodontal therapy can improve glycemic control in diabetic patients. However, only a limited number of studies have evaluated the effect of periodontitis on glycemic levels in non-diabetic individuals.^17^ Wolf et al^18^ reported that periodontitis was associated with a small increase in glycosylated hemoglobin in non-diabetic adults.

Recent studies support the evidence of a reciprocal relationship between periodontitis and diabetes mellitus.^19-20^ The relationship between periodontitis and defective metabolism of glucose has been fully demonstrated at molecular and cellular levels.^21^

Given the cellular, molecular, and biochemical effects of periodontitis on glycemic control, periodontal treatment seems to have a significant impact on diabetes control.

Although many studies have reported a link between periodontitis and diabetes mellitus, the impact of periodontitis on pre-diabetes is not clear. There is a paucity of studies in the literature that indicate fasting blood glucose levels in periodontitis.^17,22^ More evidence is available to compare HbA1c levels in healthy non-diabetic patients with periodontitis, and limited studies have evaluated the effect of periodontal treatment on HbA1c levels in non-diabetic individuals. Thus, this study compared HbA1c levels in non-diabetic patients without periodontitis and non-diabetic patients with chronic periodontitis at baseline and evaluated the effect of non-surgical periodontal treatment on glycemic control in patients with non-diabetic chronic periodontitis.

## Methods

### Study Setting

This interventional study was conducted in the Dental Clinic of Guilan University of Medical Sciences, Rasht, Iran. The study protocol was approved by the Ethics Committee of Guilan University of Medical Sciences (IR.GUMS.REC.1397.475). Based on the inclusion criteria, the study was carried out by dividing the subjects into two groups: group A (non-diabetic subjects without periodontitis) and group B (non-diabetic patients with mild to moderate periodontitis).

### Sample Size Estimation

Based on the results of a study by Perayil et al^17^ and considering the first and second type errors between 0.05 and 0.20, respectively, the sample size was estimated at n=9 for each group (taking into account a 30% dropout rate for each group of 12). In order to increase the validity of the study, 15 subjects were considered for each group.

### Inclusion criteria

#### Group A (non-diabetic subjects without periodontitis)

1. Age ≥35 and ≤65 years
2. Systemically healthy; no bleeding upon probing, probing pocket depth (PPD) ≤2 mm, and no clinical attachment loss (CAL)
3. The presence of more than 20 natural teeth
4. Fasting blood sugar (FBS) <100 mg/dL.

#### Group B (non-diabetic individuals with mild to moderate chronic periodontitis)

1. Age ≥35 and ≤65 years
2. Systemically healthy, PPD ≥3 mm, and CAL in at least 30% of areas between 1 and 4 mm
3. The presence of more than 20 natural teeth
4. LFBS <100 mg/dL.^17^

#### Exclusion criteria

1. A history of diabetes mellitus or systemic diseases
2. A history of antibiotic use or periodontal therapy within the past six months
3. Pregnancy, lactation
4. Smoking, alcohol use
5. The presence of poor restoration and prosthetic devices.^17^

The subjects were non-diabetics, aged 35‒65 years, with and without periodontitis, who reported to the Dental Clinic of the International Campus of Guilan University of Medical Science. The subjects were consecutively selected; randomization was not implemented due to the limited number of subjects who fulfilled the criteria for selection.

The subjects who fulfilled the inclusion/exclusion criteria were invited to participate in the study and divided into two groups: group A (non-diabetic subjects without periodontitis) and group B (non-diabetic subjects with periodontitis). Written informed consent was obtained from all the subjects who agreed to participate in this study.

The patients were categorized based on their sex (male/female). Questions were asked about smoking and the use of alcohol, and non-smokers and non-alcoholics were included in the study. Non-smokers were defined as those who had smoked <100 cigarettes and non-alcoholics were defined as those who had consumed <12 drinks in their lifetime.^23^

After seating the subjects on the dental chair and obtaining their consent and filling out the prepared information forms, fasting blood sugar (FBS) was measured and recorded to prove the non-diabetic status, and HbA1c level was measured and recorded to evaluate the glycemic control of the two groups. The patients had already been told that an FBS test required at least 8 hours of fasting.

To measure the levels of FBS and HbA1c, 5 mL of peripheral blood was taken with a syringe from the antecubital vein by a trained nurse in the laboratory. HbA1c sample tube contained an anticoagulant, K2EDTA. Pars Azmoon kit (Iran) and Nycocard kit (Alere, Norway) were used to evaluate FBS and HbA1c levels, respectively.

HbA1c was reported with IFCC (The International Federation of Clinical Chemistry and Laboratory Medicine) unit.^17^

A laboratory review of HbA1c for diabetes control is as follows: 4‒6%, known as non-diabetic; <7%, known as good control; 7‒8%, known as intermediate control; and >8%, known as a poor control.^24^ The FBS levels announced by the International Committee of Experts in 2018 in mg/dL are as follows: 70‒100, known as normal; 100‒125, known as pre-diabetic; ≥126, known as diabetic.^25^

After the measurement, subjects with FBS levels <100 mg/dL were included in the study.^17^

The subjects were clinically examined using a mirror and a periodontal probe ('O' Michigan, coded by Williams JUYA Pakistan) by a periodontist. The parameters probing pocket depth (PPD), plaque index (PI), gingival index (GI), and clinical attachment level (CAL) were measured and recorded.

The PI (The O'Leary Index) was calculated by dividing the number of surfaces with plaque by the total number of surfaces (the number of available teeth multiplied by 4) and multiplying it by 100 to obtain the percentage of surfaces with plaque.^26^ The GI (Loe & Silness) was used to assess gingival inflammation status.^27^

PPD and CAL were determined for all teeth using a Michigan periodontal probe ('O' with Williams JUYA-Pakistan Coding) at six zones for each tooth (mesiobuccal, midbuccal, distobuccal, mesiolingual, midlingual, and distolingual), and the longitudinal axis parallel probe was inserted into the pocket and moved around each tooth surface in a walking motion. We retrieved the numbers upwards, and the results were recorded in millimeters.

People in group A might have required non-surgical periodontal treatment due to plaque and supragingival calculus. It should be noted that the treatment was not harmful to individuals and was for periodontal health. It was also not ethically correct to maintain plaque and supragingival calculus in group A patients without appropriate therapy. Therefore, according to the above description and based on a similar study,^17^ all the subjects were treated with non-surgical periodontal therapy. Scaling and root planing were performed for each individual by a periodontist in one session for each subject with an ultrasonic device. Each session lasted approximately one hour. At the beginning of the session, the subjects received oral health instructions, and they were reminded at the end of the session to follow the instructions.

All the subjects were reassessed at the follow-up session three months later, and HbA1c levels and all the periodontal clinical parameters (GI, PI, PPD, CAL) were recalculated.^17^

### Statistical Analysis

The data were analyzed with SPSS 21, and the means and standard deviations were used for descriptive statistics. Independent t-test and one-way ANOVA were used to compare the quantitative variables studied in this study. Non-parametric tests were used in the absence of assumptions. Chi-squared test was used to examine the relationship between the quantitative variables. Statistical significance was set at P<0.05.

## Results

Group A (non-diabetic subjects without periodontitis) consisted of nine males (60%) and six females (40%), with a mean age of 36.87±4.01 years. Group B (non-diabetic subjects with mild to moderate chronic periodontitis) consisted of four males (26.7%) and 11 females (73.3%), with a meanage of 45.73±6.28 years.

**Figure 1:**
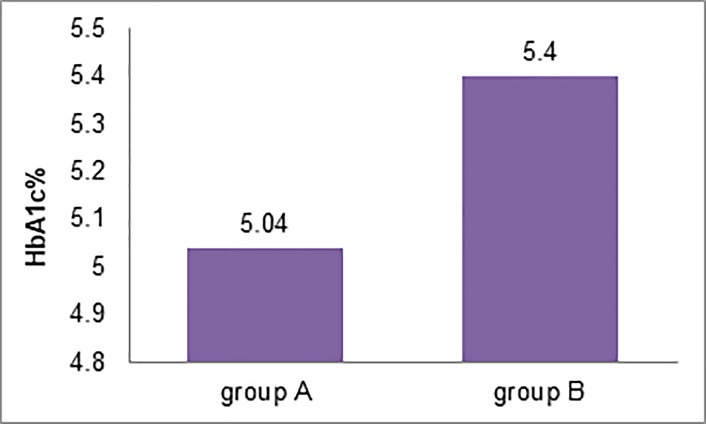
Comparison of the mean HbA1c levels in group A (non-diabetic patients without periodontitis) and group B (non-diabetic patients with mild to moderate chronic periodontitis) before non-surgical periodontal treatment (baseline).

Table 1 presents the comparison of periodontal clinical parameter values and laboratory variables of group A and group B at baseline and before non-surgical periodontal therapy. At baseline, HbA1c levels in group B (5.40±0.42) were significantly higher than those in group A (5.04±0.43) (P=0.03) (Table 1) (Figure 1). At the baseline, group B subjects had significantly higher FBS (P=0.03), PI (P=0.03), GI (P=0.001), and PPD (P=0.001) levels than the group A subjects. Group B had a CAL of 1.73±0.7 mm at baseline, while group A had no CAL (Table 1).

**Table 1:** Comparison of the periodontal clinical parameters and laboratory variables of group A (non-diabetic patients without periodontitis) and group B (non-diabetic patients with mild to moderate chronic periodontitis) before non-surgical periodontal therapy (baseline)

	**Group A** **(Mean ± SD)**	**Group B** **(Mean ± SD)**	**P-value**
**HbA1c**	5.04±0.43	5.4±0.42	0.03*
**FBS**	90.73±5.7	95.53±5.89	0.03*
**PI**	26.47±8.12	89.87±10.66	0.001*
**GI**	0.06±0.09	1.46±0.11	0.001*
**PPD**	1.00±0.00	2.33±0.48	0.001*
**CAL**	0.00±0.00	1.73±0.7	0.001*

*P<0.05 was considered significant.

Table 2 presents a comparison of the periodontal clinical parameters between groups A and B three months after non-surgical periodontal treatment.

**Table 2:** Comparison of the periodontal and laboratory clinical parameters of group A (non-diabetic patients without periodontitis) and group B (non-diabetic patients with mild to moderate chronic periodontitis) three months after non-surgical periodontal therapy

	**Group A** **(Mean ± SD)**	**Group B** **(Mean ± SD)**	**P-value**
**HbA1c**	4.98±0.43	5.13±0.41	0.33
**FBS**	89.07±6.21	94.53±5.38	0.016*
**PI**	16.93±3.51	25.4±3.43	0.001*
**GI**	0.01±0.03	0.35±0.11	0.001*
**PPD**	1.00±0.00	1.33±0.48	0.013*
**CAL**	0.00±0.00	0.33±0.48	0.013*

*P<0.05 was considered significant.

**Figure 2:**
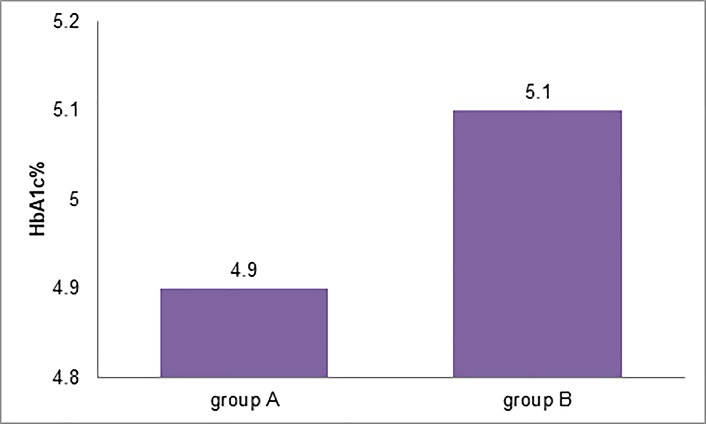
Comparison of the mean of HbA1c levels of group A (non-diabetic patients without periodontitis) and group B (non-diabetic patients with mild to moderate chronic periodontitis) three months after non-surgical periodontal treatment.

The HbA1c level three months after SRP was higher in group B (5.13±0.14) than that in group A (4.98±0.43), but the difference was not statistically significant (P=0.33) (Table 2) (Figure 2).

Three months after SRP, there was a statistically significant difference between the two groups in FBS (P=0.01), PI (P=0.001), GI (P=0.001), PPD (P=0.01), and CAL (P=0.01) (Table 2).

Table 3 shows the comparison of the periodontal and laboratory clinical parameters in group B before non-surgical treatment (baseline) and three months after treatment.

**Table 3:** Comparison of the periodontal clinical parameters and laboratory variables of group B (non-diabetic patients with mild to moderate chronic periodontitis) before non-surgical periodontal therapy (baseline) and three months after treatment

	**Before treatment**	**After treatment**	**P-value**
**HbA1c**	5.40±0.42	5.13±0.41	0.006*
**FBS**	95.5±5.89	94.53±5.38	0.001*
**PI**	89.87±10.66	25.40±3.43	0.001*
**GI**	1.46±0.11	0.35±0.11	0.001*
**PPD**	2.33±0.48	1.33±0.48	0.001*
**CAL**	1.73±0.70	0.33±0.48	0.001*

P<0.05 was considered significant.*

**Figure 3:**
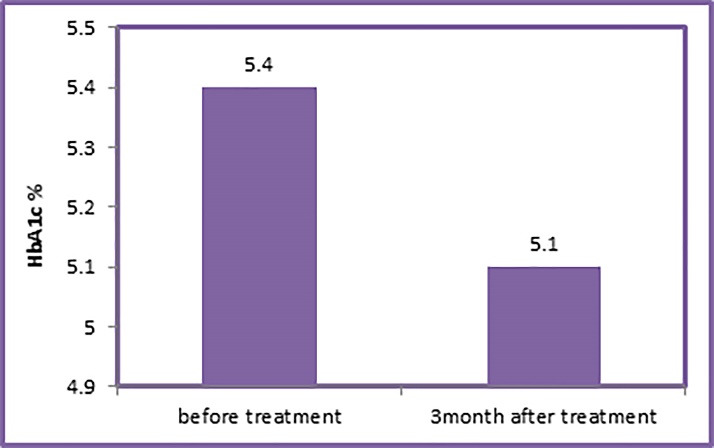
Comparison of the mean blood glycosylated hemoglobin level (HbA1c) of group B (non-diabetic patients with mild to moderate chronic periodontitis) before non-surgical periodontal therapy (baseline) and three months after treatment.

The HbA1c levels in group B (13.41±0.5) significantly decreased three months after SRP compared to the baseline (5.4±0.42) (P=0.006) (Table 3) (Figure 3).

In group B, three months after SRP, there were statistically significant reductions compared to baseline in FBS (P=0.001), PI (P=0.001), GI (P=0.001), PPD (P=0.001), and CAL (P=0.001) (Table 3).

## Discussion

This study examined the relationship between chronic periodontitis and glycemic control status in non-diabetic individuals.

Periodontitis is a conflict between pathogenic microorganisms and host defense, as the presence of periodontopathogens is essential for disease onset, but the host response to the infection is an essential factor for disease progression.

In periodontal infection, periodontal pathogens and their products increase tissue resistance to insulin and inhibit glucose entry into target cells, leading to increased blood glucose levels and worsening of glycemic control.

In the present study, the results showed that at baseline, HbA1c levels were significantly higher in non-diabetic subjects with mild to moderate chronic periodontitis compared to non-diabetic subjects without periodontitis.

Many studies, including those by Rosamma et al,^28^ Deepka et al,^29^ and Saxena et al,^30^ have compared the levels of HbA1c in non-diabetic patients with and without periodontitis and the results are consistent with those of the present study.

Many researchers have investigated the effect of periodontal non-surgical treatment on glycemic control status of diabetic patients with chronic periodontitis, and all have reported a significant decrease in HbA1c levels at follow-up.^31-33^

However, to date, few studies have investigated the effect of non-surgical periodontal treatment on the glycemic control of non-diabetic patients with chronic periodontitis.

The present study was the first study to investigate this issue in Iran and evaluate periodontal clinical parameters (PI, GI, CAL, PPD) and laboratory variables of FBS and HbA1c in non-diabetic subjects with and without periodontitis at baseline and compared it with follow-up period which was three months after non-surgical periodontal treatment.

Laboratory parameters of HbA1c and FBS levels were measured in the laboratory by a highly trained nurse with high precision, while previous studies used a glucometer and chairside kit.

Non-surgical periodontal therapy is the first step in the process of performing periodontal treatment. The goal of treating is to alter or remove microbial etiologies and factors contributing to gingival and periodontal disease. Although surgical treatments have evolved over the years, non-surgical therapy is still the gold standard.

Evaluation of the effect of non-surgical periodontal treatment has been proposed on different factors in different studies and different follow-up periods. In studies by Jayachandran et al^17^ and Jonanni et al,^34^ the follow-up period was three months. We also considered a three-month follow-up after treatment in this study because glucose in the blood is inextricably bound to hemoglobin. Glycated hemoglobin shows the glucose status during the half-life of red blood cells, which is 30 to 90 days.

The present study showed that the mean of HbA1c levels three months after non-surgical periodontal therapy in non-diabetic subjects with mild to moderate chronic periodontitis were not significantly different compared to non-diabetic subjects without periodontitis.

In the study of Jayachandran et al,^17^ however, the mean difference in HbA1c levels three months after non-surgical periodontal treatment in non-diabetic patients with chronic periodontitis and non-diabetic subjects without periodontitis was lower than the baseline. However, the difference was significant, which might be due to differences in sample size.

The present study showed that the mean of HbA1c level in non-diabetic patients with mild to moderate chronic periodontitis significantly decreased from 5.4±0.42% at baseline to 5.13±0.41% three months after treatment.

In studies by Jayachandran et al^17^ and Jonanni et al,^34^ patients with periodontitis showed improvements in all the clinical parameters and HbA1c levels significantly decreased after three months; however, this level never reached the level of healthy individuals.

Wolf et al^18^ evaluated glycosylated hemoglobin levels in patients with periodontitis and a healthy control group. The age of the subjects was considered to be >18 years. However, studies by Jayachandran et al^17^ and Jonanni et al^34^ considered the age of the study subjects between 35 and 65 years. We also included subjects 35‒65 years of age in this study, which is more appropriate because the prevalence of chronic periodontitis is higher in subjects >35 years of age, and people at this age have become more stable in their lifestyle.

Jayachandran et al^17^ and Jonanni et al^34^ evaluated body mass index of the subjects. No significant relationship was found between the body mass index and periodontitis. Although the relationship between HbA1c level and body mass index was not significant, we did not evaluate this index in the present study.

Researchers have revealed a link between periodontitis and various diseases or systemic conditions, including diabetes.^8^

In periodontitis, there is an increase in the production of proinflammatory mediators, such as IL-1β, TNF-α, IL-6, IFN-γ, and the levels of acute-phase proteins, such as CRP. All these mediators have important effects on glucose and fat metabolism. Evidence and observational studies are available on the effect of periodontal infection debridement on glycemic control in diabetic patients, and evidence suggests that periodontal infection impairs glycemic control in diabetics. Effective periodontal debridement can improve glycemic control in diabetic patients.^13-16^

Serum levels of these proinflammatory mediators will decrease after periodontal treatment. Thus, by controlling inflammation with SRP, it can reduce the deterioration of insulin resistance and reduce systemic levels of glucose and non-enzymatic glycation of hemoglobin. One of the limitations of the present study was that we did not measure serum levels of these mediators before and after SRP; however, a significant decrease in periodontal clinical parameters clearly indicates a decrease in periodontal inflammation that could lead to a decrease in HbA1c levels.

## Conclusions

Therefore, the results of the present study revealed that HbA1c levels in non-diabetic patients with chronic periodontitis significantly decreased at three-month follow-up after non-surgical periodontal treatment. However, this level did not reach the level of non-diabetics without periodontitis. It can be concluded that as the periodontal condition improves, the glycemic level reaches near-normal levels.

## Authors’ Contributions

The study was planned by AS, MN, and FN. NE carried out the interventional study. The statistical analyses and interpretation of data were made by AS. NE, FN. AS contributed to the literature review. All the authors approved the final manuscript.

## Competing Interests

The authors declare that they have no competing interests with regards to the authorship and/or publication of this paper.

## Ethics Approval

The study protocol was approved by the Ethics Committee at Guilan University of Medical Sciences (IR.GUMS.REC.1397.475).
